# Persistent fever and destructive arthritis caused by dialysis-related amyloidosis: A case report

**DOI:** 10.1097/MD.0000000000009359

**Published:** 2018-01-05

**Authors:** Kotaro Matsumoto, Jun Kikuchi, Yuko Kaneko, Hidekata Yasuoka, Kazuko Suzuki, Hirobumi Tokuyama, Kaori Kameyama, Kunihiro Yamaoka, Tsutomu Takeuchi

**Affiliations:** a Division of Rheumatology; b Division of Nephrology, Department of Internal Medicine; c Department of Diagnostic Pathology, Keio University School of Medicine, Shinjuku-ku, Tokyo, Japan.

**Keywords:** β2-microglobulin, β2-microglobulin modified by advanced glycation end products, dialysis-related amyloidosis, fever of unknown origin, long-term hemodialysis

## Abstract

**Rationale:**

Dialysis-related amyloidosis (DRA) can present rheumatic manifestations in patients on long-term hemodialysis. Typical articular symptoms with DRA involve carpal-tunnel syndrome, effusion in large joints, spondyloarthropathy, or cystic bone lesions, which are usually with non-inflammatory processes.

**Patient concerns:**

A 64-year-old man on hemodialysis for >30 years was admitted because of intermittent fever, polyarthritis, and elevated serum C-reactive protein (CRP) level, which was continuous for 2 years. Several antibiotics were ineffective for 3 months before his admission. On physical examination, joint swelling was observed at bilateral wrists, knees, ankles, and hip joints. Laboratory tests revealed elevation of serum inflammatory markers and β2-microglobulin (β2-MG). Synovial fluid showed predominant infiltration of polymorphonuclear leukocytes and the increase of β2-MG level.

**Diagnosis:**

Significant deposition of β2-MG with inflammatory cell infiltration was found in biopsied samples from synovium, skin, and ileum.

**Interventions:**

We decided to switch to the hemodialysis column with membrane that can effectively absorb β2-MG in circulation.

**Outcomes:**

The relief of symptoms and a decrease of CRP level by changing the membrane lead to the final diagnosis of DRA.

**Lessons:**

Our case demonstrates that DRA arthropathy can be inflammatory and destructive, and also develop systemic inflammatory signs and symptoms. In such cases, aggressive absorption of β2-MG in circulation might help the amelioration of symptoms.

## 1 Introduction

Amyloidosis is a condition characterized by systemic extracellular deposition of an abnormal fibrous protein, amyloid. Amyloid fibrils are formed by normal soluble proteins, which assemble to form insoluble fibers that are resistant to degradation.^[1]^ One of amyloid fibrils, β2-microglobulin amyloid (Aβ2M), are made of misfolded β2-microglobulin (β2-MG) and extracellular matrix.^[2–4]^ β2-MG in serum is usually excreted by glomerular filtration, but in patients with long-standing end-stage kidney disease who are on hemodialysis, serum β2-MG levels can be increased to up to 60 times of those in normal individuals,^[2]^ since the level of β2-MG is above the capacity of the dialyzer.^[5]^ This leads to the systemic deposition of Aβ2M, resulting in dialysis-related amyloidosis (DRA).

The typical manifestation of DRA includes carpal-tunnel syndrome, non-inflammatory effusion in large joints, spondyloarthropathy, and cystic bone lesions. However, inflammatory symptoms are usually rare and only a few cases with fever or inflammatory arthritis have been reported.^[6–8]^

Here, we report a unique case of DRA with systemic inflammation who developed persistent fever and erosive arthritis, which was improved by efficient hemofiltration of β2-MG.

## 2 Case presentation

A 64-year-old Japanese man on hemodialysis for over 30 years was admitted to our hospital in 2016 with intermittent fever, polyarthritis, and elevated C-reactive protein (CRP). He was diagnosed as end-stage kidney disease due to nephrosclerosis in 1984, and hemodialysis was initiated in 1985. He underwent bilateral carpal-tunnel syndrome surgery in 2013. In 2015, he started to feel polyarticular pain with stiffness of his bilateral shoulders, knees, and also back pain. These manifestations were gradually exacerbated. The dull pain persisted almost whole day, and increased by flexing. He did not realize the morning stiffness. Three months prior to admission, he developed fever, especially following dialysis. Several antibiotics such as ceftriaxone and levofloxacin were administered for a few weeks, but showed no effects. No other symptoms of cough, appetite loss, weight loss, or night sweats were present.

On admission, physical examination revealed a normal blood pressure of 120/70 mmHg and body temperature of 39.0 °C. His bilateral wrists, knees, ankles, and hip joints were swollen with tenderness. Flexion contracture was also observed in both hands and knees. Auscultation of heart sounds showed irregular rhythm, normal S1 and S2, and Levine III/IV systolic murmurs on from 2nd to 4th left sternal borders due to moderate to severe tricuspid regurgitation. There were no redness or tenderness on his vascular graft. No other abnormal findings upon examination of the eyes, lymph nodes, lungs, abdomen, neurological systems, and skin were observed.

**Figure 1:**
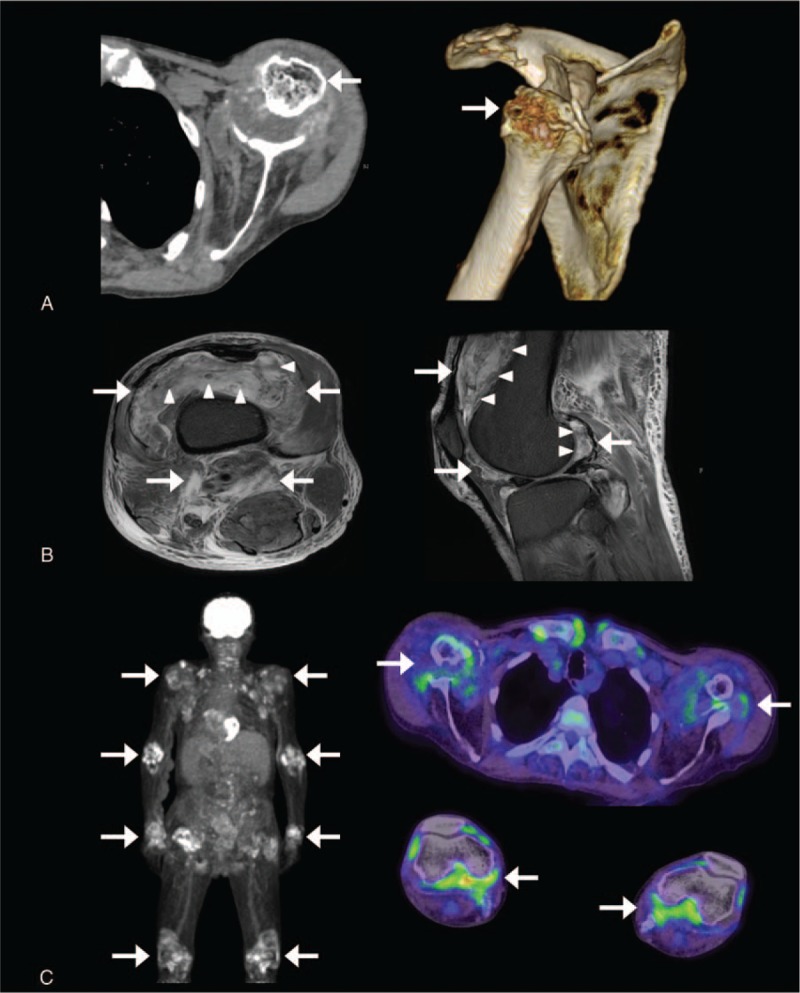
Images of CT, MRI, and 18F-FDG PET of the patients. (A) A representative image of computed tomography of right shoulder. Damaged shoulders (shown by arrows) surrounded by low-density soft tissue was shown. (B) T2-STIR magnetic resonance images of the right knee. A low-intensity area (shown by thick allow) of amyloid deposits and a high-intensity area (shown by thin allow) representing fluid collection. (C) Images of 18F-FDG positron-emission computed tomography. FDG uptake in the large joints was shown with arrows. CT = computed tomography, DRA = dialysis-related amyloidosis, FDG = fluorodeoxyglucose, MRI = magnetic resonance imaging, STIR = T2-weighted short-tau inversion recovery.

**Figure 2:**
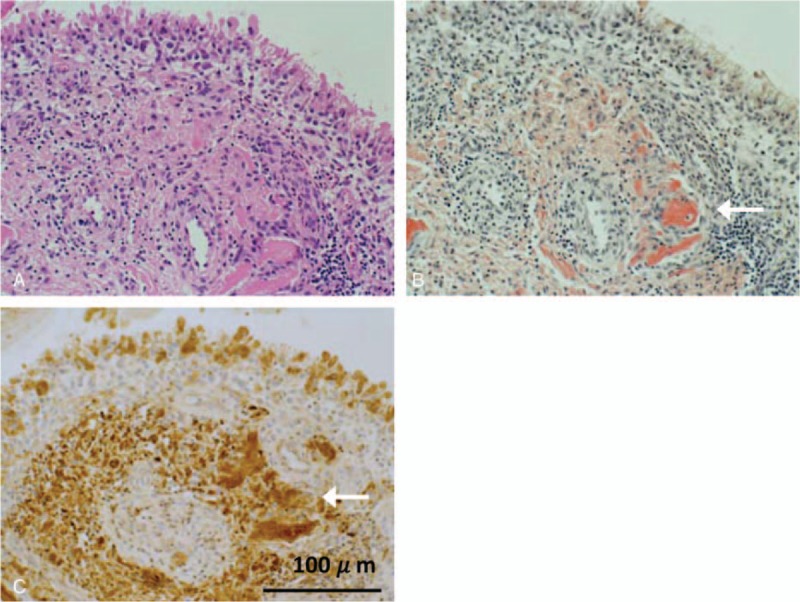
Histological images of synovium samples of the case. (A) Hematoxylin-eosin staining of the synovial tissue. Prominent lymphocytic and plasmacytic infiltration beneath enlarged synovial lining cells. Amorphous eosinophilic material depositions were seen around capillaries (arrow) (×200). (B) Congo-red staining of synovial tissue. The amorphous materials were stained orange-red (arrow) (×200). (C) β2-MG deposition of synovial tissue by immunohistochemistry. Browny staining showed deposition of β2-MG (×200). β2-MG = β2-microglobulin.

Laboratory tests of blood showed an elevated CRP (9.22 mg/dL, normal range: <0.35 mg/dL), erythrocyte sedimentation rate (124 mm/h, normal range: <15 mm/h), and matrix metalloproteinase-3 (>1200 ng/mL, normal range: 35.2–123.8 ng/mL). The serum β2-MG level was extremely high (25.68 mg/L, normal range: 0.9–1.7 mg/L). Total protein was 6.5 g/dL and no monoclonal M peak or abnormal *κ*/*λ* ratios were found (Table 1). Arthrocentsis was also examined and the cell count in his right knee was 9700/μL with predominant polymorphonuclear leukocytes. Gram staining and bacterial culture were negative, but calcium pyrophosphate dihydrate (CPPD) was detected. Moreover, β2-MG in the synovial fluid was detected with a higher concentration (34.97 mg/L) than that in peripheral blood. Computed tomography (CT) of bilateral shoulder joints, humeri, and hip joints showed severely damaged lytic bone lesions surrounded by low density soft tissue (Fig. 1A). A T2-weighted short-tau inversion recovery (STIR) magnetic resonance imaging (MRI) of the right knee joint showed a low-intensity area with fluid collection (Fig. 1B). Positron emission tomography-CT with [18F]-fluorodeoxyglucose (FDG) demonstrated abnormal FDG uptake in the large joints (standardized uptake value max: 7.6) as shown in Fig. 1C. These findings did indicate existence of destructive arthritis and abnormal periarticular deposition. Considering malignant tumor or infectious disease, we conducted biopsies from his right knee synovium, right thigh skin, and mucosa of the ileum, colon, and rectum. Histological examination of the synovium of the knee revealed prominent infiltration of lymphocytes and plasma cells beneath enlarged synovial lining cells. There were no findings of myeloma cells or CPPD crystals, and *κ*/*λ* ratio was within normal range. Amorphous eosinophilic material depositions were seen around capillaries (Fig. 2A), which were also stained as orange-red by Congo-red staining (Fig. 2B). Also, β2-MG was positively detected by immunohistochemistry (Fig. 2C). Taken together, these findings suggest Aβ2M deposition. Deposition of Aβ2M and chronic inflammation were also observed in the subcutaneous collagen tissue and iliac mucosal tissue. No evidence of heart amyloidosis was detected by transthoracic echocardiography. Although it was difficult to rule out clearly the possibility of symptomatic pseudogout, histological findings did not support the diagnosis. According to the literature, asymptomatic CPPD crystals in joint fluids could be presented in elderly patients.^[9]^ Multiple myeloma was the first differential diagnosis considering the multiple osteolytic lesion, but the examinations made the possibilities less likely. Malignancies or infectious diseases, which was considered from multiple sites of positive uptake in PET-CT, were denied by several biopsies. Bacterial infection was also denied by negative blood or synovial fluid cultures, β-D glucan, or interferon-gamma release assay. Finally, dialysis-related Aβ2M amyloidosis was suspected to cause fever.

**Table 1:**
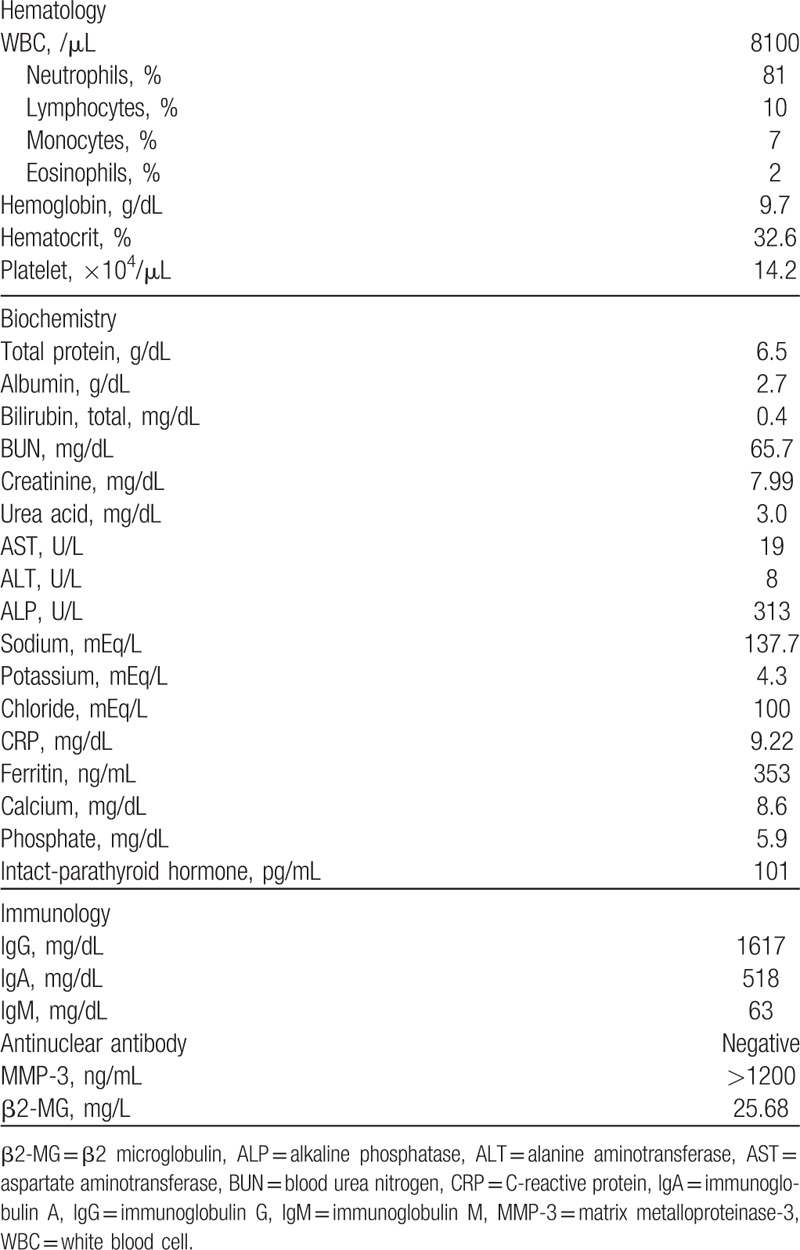
Laboratory findings.

**Figure 3:**
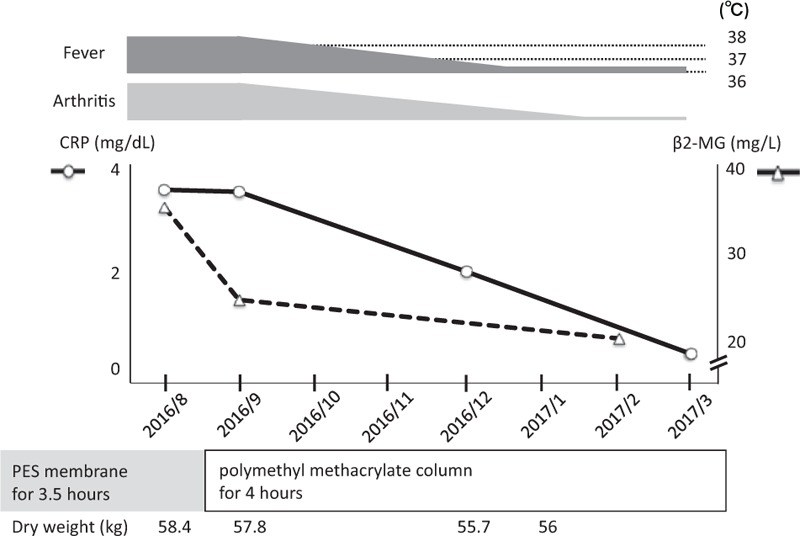
The clinical course after treatment intervention. CRP = C-reactive protein, β2-MG = β2-microglobulin, PES = polyethersulfone.

Dialyzer used for hemodialysis was polyethersulfone (PES) membrane, which has low efficiency of urea removal rate of 53% and *Kt*/*V* of 0.9, suggesting that filtration efficiency of β2-MG of hemodialysis of the patient was also relatively low. Thus, we switched to the polymethyl methacrylate column that has a superior efficiency of Aβ2M removal.^[10]^ In addition, the time of one dialysis session was extended from 3.5 to 4 hours. After the dialysis with the new column was started from August 2016, serum β2-MG, dry weight, and cardio-thoracic ratio of chest x-ray gradually decreased. Then, we finally confirmed normalization of the high levels of inflammatory marker and fever with his articular symptoms improved in January 2017 (Fig. 3). Considering the several months of delay from treatment intervention to amelioration of the disease state matched to DRA, rather than pseudogout. Taken together of these reasons, we thought the trigger of inflammation was attributed to deposition of Aβ2M.

## 3 Discussion

We showed the case with DRA presenting fever and erosive arthritis caused by the massive, long-term deposition of Αβ2M, suggesting that DRA could cause a systemic inflammatory response and destructive joint inflammation.

The risk factors that promote DRA are long-term hemodialysis treatment, elderly initiation of hemodialysis, lack of remaining renal function, and a dialysis with low filtration efficiency.^[11,12]^ The deposition of Aβ2M occurs mainly in bone, cartilage, and synovial membrane, and nerve compression symptoms such as carpal-tunnel syndrome, bone cyst, and bone destruction are often developed. The frequency of joint restriction and effusions in large and medium size joints rises along with the duration of hemodialysis.^[13]^ Although Aβ2M can also deposit in various organs and result in multiple organ disorders, gastrointestinal and cardiac involvement is rare in DRA.^[14,15]^ Our case had a long-term usage of conventional dialyzer, which had relatively low filtration efficiency but convenient and low cost, resulting in developing intensive joint symptoms, but without skin eruption or abdominal symptoms despite the microscopic deposit of Aβ2M in the skin and ileum, and no cardiac involvement.

As mentioned above, polymethylmethacrylate dialysis membranes possibly improve his arthritis due to good filtration efficiency of β2-MG. However, they remove solutes of circulating cytokines and some cationic compounds as well as β2-MG.^[16]^ We cannot deny another hypothesis that polymethylmethacrylate dialysis membranes improve inflammation by removing inflammatory compounds other than β2-MG.

The question of whether DRA might cause fever remains controversial.^[17,18]^ In the amyloid plaques of DRA patients, β2-MG is modified by advanced glycation end products (β2MG-AGE).^[19,20]^ In uremic patients, increased 3-deoxyglucosone, a highly reactive dicarbonyl compound, may promote the AGE modification of β2-MG,^[21]^ and β2MG-AGE may lead to an inflammatory response and the recruitment of monocytes and macrophages around amyloid deposits. β2MG-AGE is biologically active and interacts with mononuclear phagocytes and synovial fibroblasts through the receptors of advanced glycation end products, stimulating the release of proinflammatory cytokines for monocytes or macrophages.^[22]^ The fever often seen following dialysis, as in our case, may be explained in part by induced cytokine production and complement activation due to the dialysis treatment.^[23]^ The activation of inflammatory cascades has been attributed to the exposure of blood to dialysis membranes or the back leakage of lipopolysaccharide through the dialysis membranes.^[24]^

Although some previous cases with Aβ2M amyloidosis with systemic inflammation were reported to need glucocorticoid therapy,^[6]^ our case was treatable by dialyzer membrane exchange and an extended dialysis time, suggesting that dialysis efficiency is important in the treatment and prevention of DRA.

## 4 Conclusions

Our case highlights that DRA can lead to systemic inflammation and destructive inflammatory arthritis. Physicians should be aware of DRA as a cause of fever in a case with a long history of dialysis and a high level of serum β2-MG. Aggressive absorption of β2-MG in circulation might help the amelioration of symptoms in such cases.
